# A two-sample bidirectional Mendelian randomization analysis between telomere length and hyperthyroidism

**DOI:** 10.3389/fendo.2024.1369800

**Published:** 2025-01-22

**Authors:** Shiben Zhu, Ziyu Hao, Qihang Chen, Xiaoliu Liu, Wenyan Wu, Fang Zhang

**Affiliations:** ^1^ School of Nursing and Health Studies, Hong Kong Metropolitan University, Hong Kong, Hong Kong SAR, China; ^2^ Jockey Club School of Public Health and Primary Care, The Chinese University of Hong Kong, Hong Kong, Hong Kong SAR, China; ^3^ Medical Laboratory of Shenzhen Luohu People’s Hospital, Shenzhen, Guangdong, China; ^4^ Department of Science and Education, Shenzhen Baoan Women’s and Children’s Hospital, Shenzhen, Guangdong, China

**Keywords:** Mendelian randomization analysis, telomere length, hyperthyroidism, casual effect, GWAS

## Abstract

**Background:**

hyperthyroidism characterized by low thyrotropin, highlighting complications and risks, including cardiac issues, osteoporosis, adverse pregnancy outcomes, unintentional weight loss, and increased mortality associated with untreated hyperthyroidism. However, the casual association between telomere length (TL) and hyperthyroidism remains unclear.

**Objective:**

We aim to explore the casual relationship between TL and hyperthyroidism.

**Methods:**

A two-sample bidirectional Mendelian randomization (MR) analysis employed the inverse variance weighted (IVW) method, supplemented by additional approaches such as Weighted Median (WM), and MR Egger.

**Results:**

The summary statistics for TL were derived from the UK Biobank, comprising 472,174 individuals, while the data for hyperthyroidism were sourced from the GWAS Catalog and the FinnGen database, encompassing cohorts of 460,499 and 173,938 individuals, respectively. Utilizing 139 genome-wide significant single nucleotide polymorphisms (SNPs) as instrumental variables (IVs) for TL, forward MR analyses indicated a negative causal effect of TL on hyperthyroidism. The risk of hyperthyroidism decreased as genetically predicted TL increased by one standard deviation, as determined by the IVW form GWAS Catalog (OR:0.659,95%CI: 0.541-0.802, *p <*0.001) and IVW from FinnGen(OR:0.634, 95%CI: 0.479-0.840, *p* = 0.001). Other MR methods exhibited a consistent trend in the impact of TL on hyperthyroidism. Reverse MR analysis suggested no causal association between TL and hyperthyroidism (*p* > 0.05). Sensitivity analyses confirmed the robustness of these results, suggesting minimal susceptibility to confounding factors and bias.

**Conclusion:**

The finding that longer telomeres reduce hyperthyroidism risk highlights the need to validate hyperthyroidism’s impact on telomere length, offering valuable insights for prevention and treatment.

## 1 Introduction

Hyperthyroidism, a prevalent endocrine disorder, affects 0.2-1.3% of the general population (1), with women more affected than men, and increases with age (2). Given the critical role of thyroid hormones in essential physiological processes like growth (3), maturation (4), and metabolism (5), many efforts have the positive association between thyroid function and cancer development (6, 7). Without treatment, hyperthyroidism can lead to serious complications including cardiac arrhythmias (8), congestive heart failure (9), osteoporosis (10), adverse obstetric outcomes (11), and metabolic imbalances (12), such as increased resting energy demand and gluconeogenesis (13). The intricate consequences of hyperthyroidism highlight the importance of understanding its prevalence and the diverse underlying mechanisms, emphasizing the need for effective control and prevention of associated disorders (14).

Hyperthyroidism, characterized by elevated thyroid hormone levels, plays a significant role in the aging process. Age-related changes in thyroid function have important implications for longevity (3). Studies suggest that longevity in vertebrates is positive associated with low metabolic rates and TH levels (15), and thyrotoxicosis in mice has been linked to aging traits like malnutrition and immune senescence (16). Telomeres are indispensable DNA-protein complexes at the ends of chromosomes, crucial for maintaining genomic stability by protecting repeated “TTAGGG” sequences (15). However, with each cell division, telomeres shorten, leading to replicative senescence, genetic instability, and ultimately cell death when critically short (16). Telomere length (TL) has been extensively studied as a biomarker for human aging across various tissues (17), with research linking TL to increased susceptibility to conditions such as cardiovascular disease (18), type 2 diabetes (19), cancers (20), Alzheimer’s disease (21), chronic kidney disease (22), chronic obstructive pulmonary disease (23), and alcohol consumption (24). However, the casual relationship between TL and hyperthyroidism remains underexplored.

Mendelian randomization (MR) as a statistical tool is used in epidemiology to examine causal associations between exposures, biomarkers, or risk factors and outcomes. MR is particularly useful in situations where conducting randomized controlled trials is not feasible or poses ethical dilemmas (17). MR is an excellent approach for mitigating the issues of residual confounding and reverse causality, which are sometimes encountered when studying observational data using alternative approaches (18). Recent research has explored the connections between depression (19), mortality (20), Graves’ diseases (21), multiple sclerosis (22), and TL. However, no MR analysis investigated the causal relationship between TL and hyperthyroidism.

Our study investigates the causal relationship between TL and hyperthyroidism using a two-sample bidirectional Mendelian randomization analysis. Using genome-wide association study (GWAS) data, we present casual evidence that genetically predicted longer TL decreases the risk of developing hyperthyroidism. Then, we validate these findings with an independent dataset from the Finne cohort. These findings underscore the potential of telomere length as a biomarker for hyperthyroidism, offering insights that could inform novel preventive and therapeutic strategies, including personalized treatments.

## 2 Methods

### 2.1 Study design

**Figure 1:**
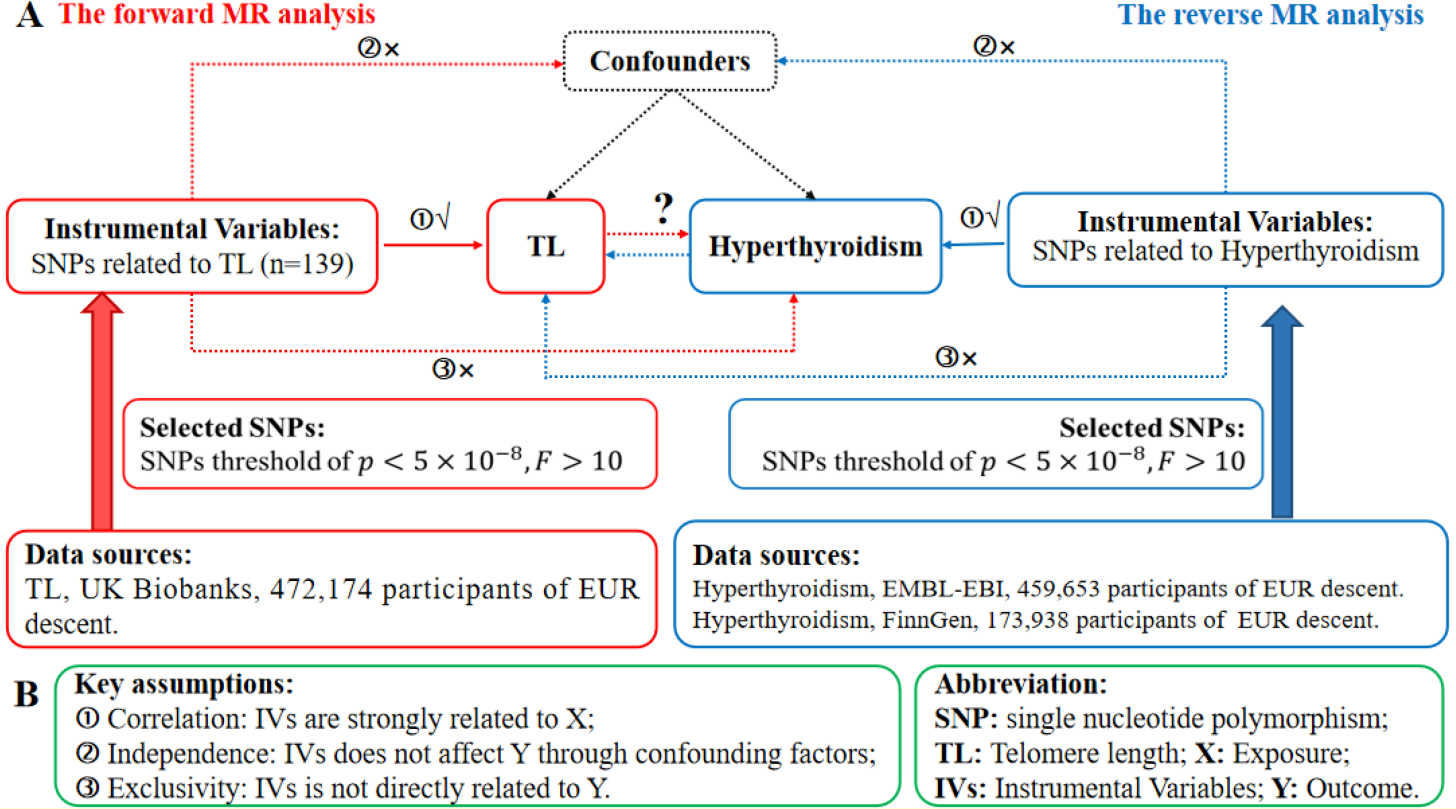
The research design in the bidirectional MR analysis. **(A)** Schematic illustrating the experimental plan. Red represents the use of forward MR analysis, where TL is used as the predictor and hyperthyroidism is the outcome. The color azure represents the use of reverse MR analysis, where hyperthyroidism is used as the predictor and TL as the result. **(B)** The three essential assumptions of MR analysis. SNPs refer to single nucleotide polymorphisms, IVs are instrumental variables, TL stands for telomere length, MR represents Mendelian randomization, X denotes the exposure, and Y represents the outcome.

We perform a conventional bidirectional MR study, using single nucleotide polymorphisms (SNPs) that are strongly linked to the target variable as instrumental variables (IVs). The GWAS datasets were used to assess the probable causal impact of the exposure on the outcomes. Genetic variants are the primary and effective IVs in a MR study. To be considered qualified IVs, they must adhere to three fundamental principles specified in MR theory. **Figure 1A** depicts the whole flowchart of this MR study, whereas **Figure 1B** represents the basic MR assumptions and acronyms. Furthermore, the data for exposure and outcomes were obtained from separate and independent samples.

### 2.2 Selection of IVs

To discover SNPs substantially linked with both TL and hyperthyroidism, we used a strict significance criterion of *p* < 5 × 10^-8^. In addition, we used strict criteria to exclude any association between genetic markers, using a 10,000 kilobase aggregation window and putting the *r* ^2^ threshold at 0.001. Afterwards, each SNP was carefully examined for any departures from basic assumptions ② and ③ by consulting the PhenoScanner database. To evaluate the efficacy of IVs, we calculated the *F*-statistic for each SNP as well as for the full set. We take the formulas (23, 24) for calculating *F*-statistic for single SNP and total set. A *F*-statistic greater than 10 suggests a significant association between the SNP and the observed phenotype.

MR also entails ascertaining the concordance between the exposure SNP and its impact on the same gene, therefore influencing the outcome. We excluded palindromic SNPs to prevent any potential biases caused by strand orientation or allele coding. We removed palindromic SNPs with intermediate allele frequency and standardized the exposure and outcome data. The MR-PRESSO (25) and MR Egger (26) methods were used to mitigate the impact of horizontal pleiotropy. The MR-PRESSO outlier test generated *p*-values to assess the pleiotropy of each SNP, while the global test determined an overall *p*-value to quantify horizontal pleiotropy. The SNPs were sorted based on their MR-PRESSO outlier test *p*-values. Subsequent global testing of MR-PRESSO was conducted on the remaining SNPs after the elimination of each individual SNP. The *p*-value surpassed 0.05, indicating a lack of statistical significance. The subsequent MR study used the remaining SNPs after excluding pleiotropic SNPs. Then inverse MR analysis has been finished.

### 2.3 Data source

All data was obtained from the public available IEU Open GWAS project. After a thorough and careful evaluation, we excluded unnecessary studies and individuals who were not of European descent. Our analysis utilized summary-level data from GWAS that specifically investigated the genetic factors linked to TL. The TL data we primarily relied on came from the UK Biobank (27). More precisely, genetic variations associated with the length of telomeres were obtained from GWAS that included a group of 472,174 people. This group included an almost equal proportion of men (45.8%) and females (54.2%), and all participants were of European descent (28). In the case of hyperthyroidism, SNPs were chosen as IVs from a GWAS dataset obtained from the GWAS Catalog (29) and FinnGen. The sample size of Hyperthyroidism which from the GWAS database consists of 460,499 people of European descent, consisting of 3,557 cases and 456,942 controls. The sample size of Hyperthyroidism which from the FinnGen consists of 173,938 people of European descent, consisting of 962 cases and 172,976 controls. **Table 1** presents a concise summary of the results in this investigation.

**Table 1:** An overview of the GWAS summary statistics.

Traits	Data source	Author & Year	Sample Size	Cases	Control	No. of SNPs	Sex	Ancestry	GWAS ID
TL	UK Biobank	Codd et al. (2021)	472,174	0	472,174	20,134,421	Males and Females	European	ieu-b-4879
Hyperthyroidism	GWAS Catalog	Sakaue et al. (2021)	460,499	3,557	456,942	24,189,279	Males and Females	European	ebi-a-GCST90018860
Hyperthyroidism	FinnGen	Tuomo Kiiskinen et al. (2021)	173,938	962	172,976	16,380,189	Males and Females	European	finn-b-AUTOIMMUNE_HYPERTHYROIDISM

### 2.4 Statistical analysis

In order to examine the cause-and-effect connection between TL and hyperthyroidism, we used the IVW random-effects model (30) as our main method. Combining Wald for each SNP within a meta-analysis paradigm was part of this analysis. Additionally, we utilized other two techniques, such as the WM (31), MR Egger (26) to reassure that our results would remain stable. We conducted sensitivity analyses to ensure robustness, including assessments for heterogeneity, pleiotropy, leave-one-out tests, and MR-PRESSO analysis. Heterogeneity was assessed with Cochran’s Q test (32) (< 0.05 considered significant). MR-Egger intercept analysis evaluated directional pleiotropy (*p* > 0.05 indicates negligible pleiotropy). MR-PRESSO assessed outliers. Leave-one-out tests confirmed stability and reliability of causal relationships. The MR analyses were performed using the TwoSampleMR, mr.raps, forestploter, and MR-PRESSO programs in the R statistical environment, version 4.3.2.

## 3 Results

### 3.1 Instrumental variables

In our forward MR, we originally chose 154 SNPs as IVs to investigate the relationship between TL and hyperthyroidism. PhenoScanner analysis could not find any connections between these SNPs and established confounding factors or outcomes. After removing palindromic SNPs, there were 141 remaining SNPs that were suitable for study. During the MR-PRESSO outlier testing, in the GWAS database, two SNPs, namely rs10774624 and rs2763979, were detected as outliers. In FinnGen, two SNPs, namely rs2306646 and rs762810, were detected as outliers. Post-outlier removal, every single SNP in this revised group had an F-statistic more than 10, thereby leading to a *R* ^2^ value of 3.72%, with the combined F-statistic reaching 120.

In the reverse MR analysis, we first selected 13 SNPs as instrumental factors for hyperthyroidism, with a specific emphasis on studying hyperthyroidism. The PhenoScanner inquiry, like the TL analysis, discovered no correlation between these SNPs and any confounding variables or outcomes. After eliminating palindromic SNPs, a total of 12 and13 SNPs remained for further study. MR-PRESSO verified the dependability of these SNPs, identifying four SNPs (rs1794280, rs2160215, rs4338740, rs758778) and three SNPs (rs11571297, rsrs11646791, rs1794511) as anomalies. After removing these outliers, every single one of these SNPs had an F-statistic that significantly exceeded 10. The aggregate coefficient of determination (*R* ^2^) for these SNPs were 8.92 and 0.621, while their cumulative F-statistic amounted to -57625 and 13.12.

A detail list of SNPs that were excluded from the final analysis owing to not satisfying the inclusion criteria can be found in **Supplementary Table S1**. The complete details of all SNPs included in the final analysis can be found in **Supplementary Table S2**, which is included in the supplementary materials.

### 3.2 MR results

**Figure 2:**
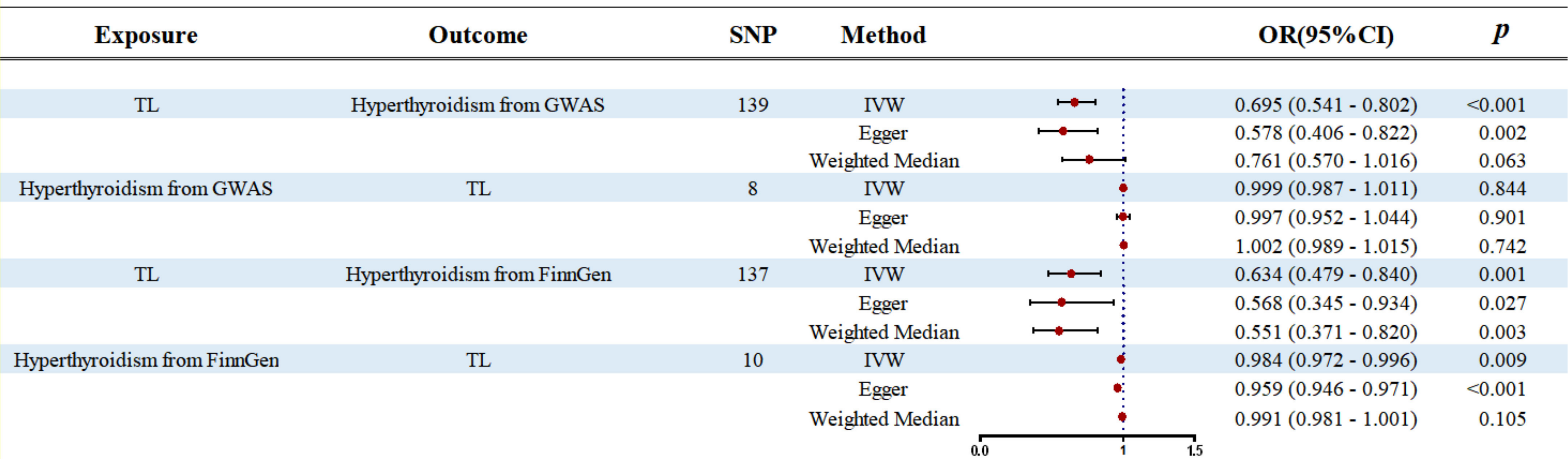
Forest plot of the bidirectional two-samples MR analysis. SNP, single nucleotide polymorphism; OR, odds ratio.

Our forward MR analysis indicates that genetically predicted increases in TL significantly reduce the risk of hyperthyroidism. This causal relationship is consistently supported by three analytical methods—IVW, Egger, and WM—as shown in **Figure 2**. In the GWAS database, IVW analysis reveals a significant negative association between TL and hyperthyroidism (OR: 0.695, 95% CI: 0.541–0.802, p < 0.001), which is corroborated by Egger (OR: 0.578, 95% CI: 0.406–0.822, p = 0.002) and WM (OR: 0.761, 95% CI: 0.570–1.016, p = 0.063). Similarly, in the FinnGen database, IVW confirms this association (OR: 0.634, 95% CI: 0.479–0.840, p = 0.001), supported by Egger (OR: 0.568, 95% CI: 0.345–0.934, p = 0.027) and WM (OR: 0.551, 95% CI: 0.371–0.820, p = 0.003).

In contrast, reverse MR analysis finds no significant association between hyperthyroidism and TL. In the GWAS database, IVW reports an OR of 0.999 (95% CI: 0.987–1.011, p = 0.844), Egger an OR of 0.997 (95% CI: 0.952–1.044, p = 0.901), and WM an OR of 1.002 (95% CI: 0.989–1.015, p = 0.742). Similarly, in the FinnGen database, IVW suggests a slight association (OR: 0.984, 95% CI: 0.972–0.996, p = 0.009), but Egger (OR: 0.959, 95% CI: 0.946–0.971, p < 0.001) and WM (OR: 0.991, 95% CI: 0.981–1.001, p = 0.105) do not indicate a strong causal link. These findings are summarized in **Figure 2**.

**Figure 3:**
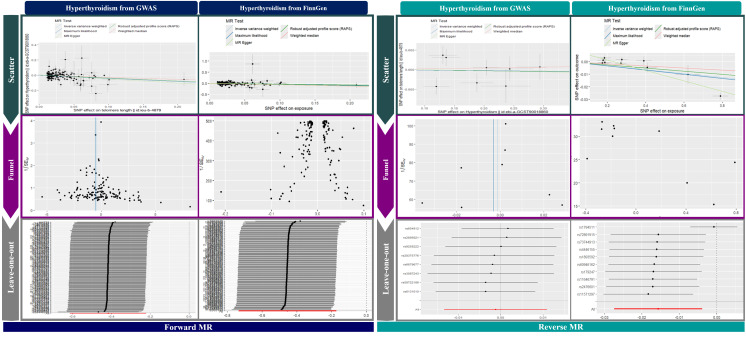
Visualization of MR analysis.

In our bidirectional MR analysis, leave-one-out evaluations and funnel plots were used to examine the relationship between TL and hyperthyroidism, as shown in **Figure 3**. The forward MR funnel plot revealed a symmetrical distribution, supported by IVW (p < 0.001, p = 0.001) and MR-Egger (p = 0.002, p = 0.027) analyses. In contrast, the reverse MR funnel plot showed asymmetry, suggesting potential bias, with GWAS IVW (p = 0.844), MR-Egger (p = 0.901), FinnGen IVW (p = 0.009), and MR-Egger (p < 0.001) results confirming this.

Leave-one-out analyses in **Figure 3** validated the stability of the results, showing a consistent negative causal link in forward MR and no significant relationship in reverse MR. Scatter plots in **Figure 3** further illustrate a significant negative association in forward MR and no correlation in reverse MR, reinforcing the robustness of our findings.

### 3.3 Sensitivity analysis

We investigated both the presence of heterogeneity and horizontal pleiotropy, and detailed findings are provided in **Table 2**. In this study, we explored the association between TL and hyperthyroidism. Utilizing 139 SNPs and 137SNPs as explanatory variables, we accounted for 3.72% and 0.9% of the variation (*r* ^2^), demonstrating robust F-statistic of 120 and 125.64. Heterogeneity tests, such as MR Egger and IVW from GWAS, resulted in Q values of 189 (*p* = 0.002) and 190 (*p* = 0.002) respectively, indicating substantial heterogeneity. In FinnGen, resulted in Q values of 208 (*p*<0.001) and 209 (*p*<0.001) respectively, indicating substantial heterogeneity. The assessment of Pleiotropy by MR Egger and MR-PRESSO indicated that the results were statistically significant.

**Table 2:** Heterogeneity and pleiotropy in our bidirectional MR analysis.

Exposure	Outcome	No. of SNPs	*R^2^*	F-statistic	Heterogeneity				Pleiotropy			
					MR Egger		IVW		MR-Egger		MR-PRESSO	
					Q	*p*-value	Q	*p*-value	Intercept	*p*	RSSobs	*p*
TL	Hyperthyroidism from GWAS	139	0.037	120	189	0.002	190	0.002	0.004	0.379	193	0.002
Hyperthyroidism from GWAS	TL	8	8.92	-57625	11.8	0.067	11.8	0.108	<0.001	0.939	14.3	0.156
TL	Hyperthyroidism from FinnGen	137	0.009	125.64	208	<0.001	209	<0.001	0.003	0.598	213	<0.001
Hyperthyroidism from FinnGen	TL	10	0.621	13.12	15.306	0.053	56.025	<0.001	0.009	0.001	120.441	<0.001

Our reverse MR study, which used hyperthyroidism as the independent variable and included 8 and 10 SNPs, produced the *R* ^2^ value of 8.92 and 0.621. F-statistic are of -57625 and 13.12. The observed direction exhibited heterogeneity, as shown by Q values of 11.8 (*p* = 0.067) and 15.306(*p* = 0.053) for MR Egger, 11.8 (*p* = 0.108) and 56.025(*p*<0.001) for IVW. The results in the GWAS database showed a lack of pleiotropic effects. in the FinnGen database, a significant pleiotropic effect was shown.

## 4 Discussion

By two-sample bidirectional MR analyses, we firstly have explored that increased telomere length is associated with decreased risk of hyperthyroidism. An previous investigation (33) suggested an association between genetic variants governing TL and thyroid cancer risk. Our current study expands upon this assertion by leveraging data from larger cohorts than previously employed. Through a comprehensive and focused analysis, we establish a causal relationship between TL and hyperthyroidism. Our findings demonstrated robustness and consistency across various MR methods. Collectively, the result offers compelling evidence supporting a causal connection between shortened telomeres and hyperthyroidism, hinting at distinct underlying mechanisms for the disease. We further validated the causal link between hyperthyroidism and TL, we found no significant association in GWAS (IVW, OR = 0.999, *p* = 0.844, 95% CI: 0.987-1.011). In FinnGen, we found association between TL and hyperthyroidism (IVW, OR = 0.984, *p* = 0.009, 95% CI: 0.972-0.996). This suggests a specific correlation between TL and the progression of hyperthyroidism. In our MR study, we incorporated valid IVs from the latest and most extensive GWAS database of TL. Subsequently, we established stringent criteria for IV selection, opting only for TL variants significantly associated with TL measurements and meeting the three core assumptions of MR analysis. Additionally, to mitigate bias in causal estimation, we employed three MR methods, ensuring the validity and consistency of results in sensitivity analyses. Addressing heterogeneity, horizontal pleiotropy, and outliers, our findings suggest that hyperthyroidism may result from shortened TL and a senescent immune system. Conversely, hyperthyroidism appears to have no impact on TL.

Hyperthyroidism itself is not typically considered an age-related disease, but the risk factors and underlying causes can be influenced by age-related factors (34). This is an intricate biological process that may be roughly categorized into replicative senescence, caused by inherent cellular mechanisms such as telomere shortening, and cellular senescence, which can be induced by different stressors such oxidative stress and DNA damage (35). Based on our research, we hypothesize that the process of telomere shortening may play a significant role in causing cellular senescence in hyperthyroidism. This is similar to how telomere-associated driver mutations are linked to rheumatoid arthritis (36), systemic sclerosis (37), and systemic lupus erythematosus (38). Shorter TL could also result from immune senescence (39). Currently, there is a growing recognition of the correlation between telomere and hyperthyroidism. The process of telomere length shortening is accelerated by age (40), and this is likely a component of the modified immune response seen in individuals with hyperthyroidism. To reduce the role of confounding factors, we excluded TL variations in our MR analysis that are related with features that might independently affect the risk of hyperthyroidism. prior research consistently indicates that a shorter TL is associated with an increased risk of developing immune-mediated inflammatory diseases (41, 42). Our results align with these prior studies.

The result indicating a cause-and-effect relationship between premature telomere erosion and hyperthyroidism offers many possibilities. Possible therapeutic strategies may include interventions targeting inadequate telomere maintenance, either on a broad scale or specifically in cells that play a significant role in the development of the illness (43). Replenishing telomere length is a complex process that cannot be easily achieved, since just enhancing the activity of telomerase may raise the likelihood of developing cancer (44). Current clinical studies, such as NCT04110964 (45), are examining this method in different medical diseases. Safe telomerase activation treatment is being investigated in other medical disciplines, such as cardiology, to mitigate the possible danger of increasing endogenous telomerase activity (46). This therapy involves temporarily delivering modified TERT RNA to prevent sustained elevation of telomerase activity (47). Despite encountering difficulties in achieving *in vivo* targeted distribution, the use of this method in hyperthyroidism may become possible in the future due to potential advancements. Androgens may also normalize telomerase levels in cells from individuals with telomere illness who have heterozygous mutations in the TERT gene (48).

Approaches that enhance general well-being while positively influencing TL promotion exist. Speculatively, these approaches could be further integrated into the clinical management of hyperthyroidism patients (49), incorporating strategies such as exercise (50), stress reduction (51), and mindfulness (52). Our findings indicate that individuals with hyperthyroidism tend to engage in reduced physical activity, likely exacerbated by poor physical health. Thoughtfully increasing exercise could yield multiple benefits, promoting chromosomal telomere length while enhancing fitness and mental health (50). Similarly, stress resulting from challenging life circumstances, like social deprivation, may be alleviated through mindfulness practices (51). Relatively straightforward measures that enhance patient well-being could fundamentally contribute to reducing telomere attrition.

Our research has many limitations. The major investigations of TL and hyperthyroidism GWAS solely involved individuals of European ethnicity. As a result, the generalizability of our findings to other populations is uncertain, emphasizing the need for stratification by various racial groups. Secondly, our MR analysis was confined to summary-level statistics, with individual-level data remaining inaccessible. This limitation restricts our ability to conduct stratified analyses based on specific factors. Thirdly, information on the subtype and severity of hyperthyroidism was not available, preventing the estimation of the relationship between TL and different hyperthyroidism subtypes and severity levels. Fourthly, there may be unidentified confounders influencing the associations between TL and hyperthyroidism that require further investigation. Lastly, it is essential to recognize that telomere length is determined by a combination of genetics, environmental factors, lifestyles, and epigenetic modifications. Hence, it is essential to acknowledge that our findings only partly clarify the causative impact of TL on hyperthyroidism.

## 5 Conclusion

The finding that longer telomeres reduce hyperthyroidism risk highlights the need to validate hyperthyroidism’s impact on telomere length, offering valuable insights for prevention and treatment.

## Data Availability

The original contributions presented in the study are included in the article/**Supplementary Material**. Further inquiries can be directed to the corresponding author.
